# Pulmonary valve replacement—A 10-year single-center surgical experience in ACHD patients

**DOI:** 10.1371/journal.pone.0310700

**Published:** 2024-10-04

**Authors:** Armin Darius Peivandi, Sven Martens, Anaïs Gion, Andreas Rukosujew, Sabrina Martens

**Affiliations:** Department of Cardiothoracic Surgery, University Hospital Muenster, Muenster, Germany; BSMMU: Bangabandhu Sheikh Mujib Medical University, BANGLADESH

## Abstract

Large-scale analyses of surgical outcomes after surgical pulmonary valve replacement (sPVR) as part of re-do surgery in adults with congenital heart disease (ACHD) are rare. Therefore, we present our outcomes of sPVR in ACHD patients over the last decade and demonstrate our standardized surgical approach. All ACHD patients who underwent sPVR between January 2013 and August 2022 were included. Primary diagnoses, peri-operative data, post-operative echocardiography, pre- and post-operative RV MRI and in-hospital mortality were examined. Pre- and postoperative MRI parameters were compared using paired testing. Standardized surgery was documented. Normality of continuous variables was tested using Shapiro-Wilk test. 79 patients (male 59.5% (n = 47), 71 re-operations (89.9%)) at a median age of 41.7 (52.2–28.8) years were included. Main underlying disease was Tetralogy of Fallot (TOF; n = 47, 59.5%). After removal of degenerated valve/conduit parts, right ventricular outflow tract (RVOT) patch augmentation and implantation of a larger stented bioprosthesis (25mm in 78.5%) were conducted. In 57% of cases, concomitant surgery was performed (mainly tricuspid valve surgery: n = 28, 35.4%). 25 patients (31.6%) were operated with beating heart technique. Echocardiographic outcomes showed no moderate or severe insufficiency (median V_max_ of 2 m/s (2.3–1.77 m/s)) upon discharge. Available MRI data showed significantly lower indexed RV-EDV (p = 0.0006) and RV-ESV (P = 0.0017) after surgery. In-hospital mortality was 5.1% (n = 4). SPVR is a safe therapeutic option with low surgical risk and satisfying post-operative results. It can serve as a solid therapeutic option for patients who need future valve-in-valve interventions.

## Introduction

Pulmonary valve procedures in adults are rare and mainly affect adults with congenital heart disease (ACHD). Overall mortality rate of such procedures currently lies at 3.9% [1].

According to present literature, clinical outcomes of surgical pulmonary valve replacement (sPVR) in ACHD patients as part of redo surgery after TOF- correction shows low mortality rates as well as low needs for re-intervention [2, 3].

Prospective magnetic resonance imaging (MRI) investigations have shown that sPVR immediately initiates reverse remodeling processes. They continue for at least 3 years after surgery. This leads to an improvement in symptoms and a reduction of right ventricular (RV) volume [4]. In this regard, the right timing of surgery is essential for a successful remodeling process after sPVR [5].

The aim of this study was to analyze the clinical data of all ACHD patients who received sPVR at our institution during the last decade.

## Material and methods

In this study, we analyze our operative data on sPVR in ACHD patients of the last decade retrospectively. In addition, we present our standardized surgical technique in this group of patients. Furthermore, available MRI and echocardiographic data is assessed.

We analyzed the clinical data of all ACHD patients who received sPVR at our institution from 01–2013 (mm-yyyy) to 08–2022 (mm-yyyy) retrospectively. Data was accessed for research purposes from 24-02-2023 (dd-mm-yyyy) to 09-04-2024 (dd-mm-yyyy). Primary diagnoses as well as peri-operative data, available pre- and post-operative RV MRI data, post-operative echocardiography and in-hospital mortality are shown. In addition, this study includes the introduction of a standardized approach for sPVR.

### Statistical analysis

Patient characteristics and results were reported as either n (%) or median (IQR 3–1). Normality of continuous variables was assessed using Shapiro-Wilk test. Paired tests (Wilcoxon test, paired t-test) were used for statistical comparison of pre- and postoperative RV MRI data. Kaplan-Meier was used for medium- and long-term follow up analysis. MedCalc V 22.009 (MedCalc Software Ltd, Ostend, Belgium) was used for statistical analysis.

### Ethical approval

The local ethics committee provided ethical approval for the retrospective analysis of ACHD patients undergoing sPVR (“Ethikkommission Westfalen-Lippe”, 2022-837-f-S). Due to the retrospective study design, informed consent of each individual was not needed and therefore waived.

## Results

### Participants

After analyzing the data of all patients above the age of 18, who received sPVR at our institution at the given time period, 80 patients were identified. One patient presented with endocarditis without a history of congenital heart disease. This patient was excluded from the study.

Hence, 79 ACHD patients were included in our retrospective analysis. 59.5% (n = 47) of them were male. Median age was 41.7 (52.2–28.8) years. 71 patients (89.9%) had undergone previous surgery, mainly during childhood. 10 patients (12.7%) had undergone balloon valvuloplasty prior to surgery.

91.1% of the surgeries were elective in nature and only in 7 (8.9%) cases there was an urgent indication for operation. Main underlying congenital heart disease was TOF (n = 47, 59.5%). Main primary intervention was fallot correction (n = 39, 49.4%). Detailed patients’ characteristics are depicted in Table 1.

**Table 1:** Patient characteristics.

Patient Characteristics	n (%) or median (IQR3-1)
Patients included	79 (100)
Male sex	47 (59.5)
Age [years]	41.7 (52.2–28.8)
Functional status (NYHA class)	
NYHA I	14 (17.7)
NYHA II	39 (49.4)
NYHA III	25 (31.6)
NYHA IV	1 (1.3)
Patients previously operated on	71 (89.9)
Prior balloon valvuloplasty	10 (12.7)
Urgent operation	7 (8.9)
Primary heart diseases	
Tetralogy/Pentalogy of Fallot;	47 (59.5)
Pulmonary stenosis	8 (10.1)
Aortic valve disease	5 (6.3)
DORV	3 (3.8)
TGA	2 (2.5)
Other	14 (17.7)
Types of primary intervention	
Fallot correction	39 (49.4)
Balloon-valvuloplasty	5 (6.3)
Ross	4 (5.1)
Aorto-pulmonary shunt	3 (3.8)
DORV correction	3 (3.8)
Waterston-Cooley-Anastomosis	3 (3.8)
BT-shunt	2 (2.5)
Commisurotomy	2 (2.5)
Other	11 (13.9)
Unknown	2 (2.5)
None	5 (5.1)
Triggers for intervention	
Pulmonary stenosis	8 (10.1)
Pulmonary insufficiency	48 (60.8)
Combined	23 (29.1)

### Pre-operative data and echocardiography

Main trigger for intervention was pulmonary insufficiency (n = 48, 60.8%), followed by combined pulmonary vitia (n = 23, 29.1%) and isolated pulmonary stenosis (n = 8, 10.1%).

Insufficiency was characterized as low grade in 6 patients (7.6%). 8 patients displayed moderate pulmonary insufficiency (10.1%), 3 had moderate-severe insufficiency (3.8%) and 54 patients (68.4%) had severe pulmonary insufficiency. In patients with pulmonary stenosis component present, 1 patient showed relative stenosis (1.3%), 4 patients showed low grade stenosis (5.1%), 2 patients had low to moderate stenosis (2.5%), 10 patients had moderate stenosis (12.7%) and 14 displayed high grade pulmonary stenosis (17.7%).

Median pre-operative maximum velocity of the pulmonary valve (V_max_) was 2.2 m/s (3.4–1.8 m/s).

### Standardized surgical approach

Depending on pre-operatively assessed vulnerability (chest computed tomography (CT) or magnetic resonance imaging (MRI)), either peripheral cannulation or central cannulation after sternotomy was performed.

**Fig 1:**
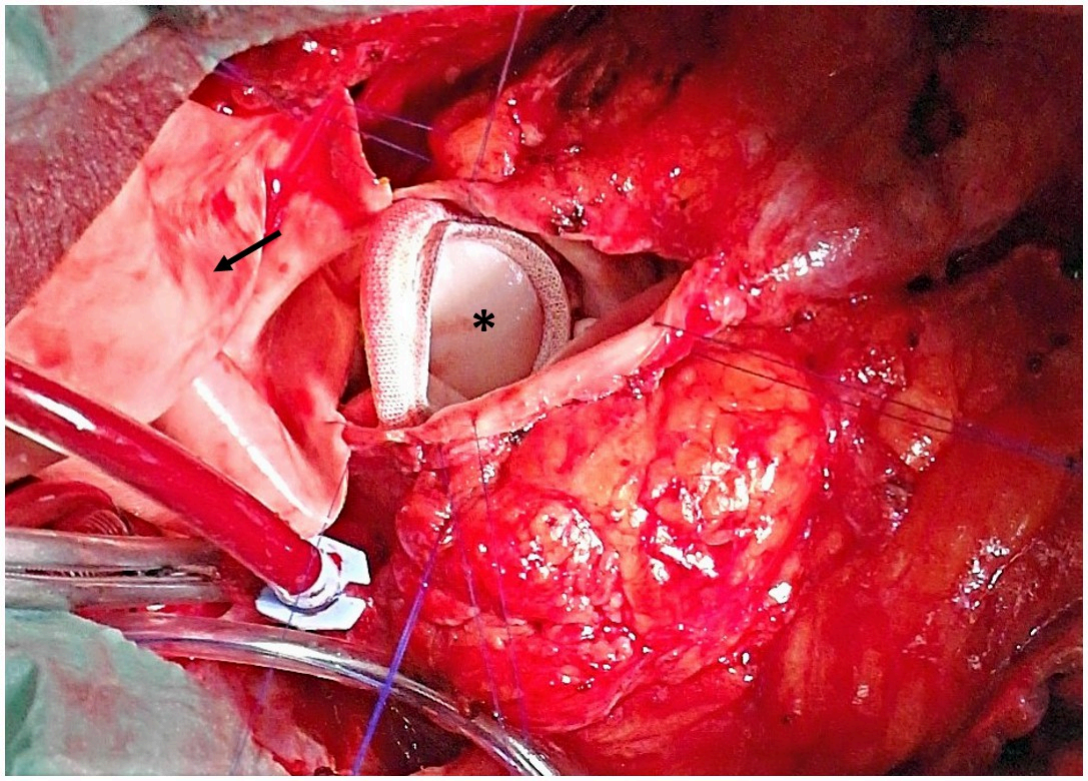
Implantation of a large diameter stented bioprosthesis (Perimount, marked with asterisk) in pulmonary position with patch enlargement of the RVOT (marked with arrow) (surgeons’ view).

Taking into account individual adaptations depending on previous operations and surgical preference, the general surgical approach can be described as follows:

After re-entry of the RVOT, possibly degenerated patch material with remnant parts of the native pulmonary valve or the destructed conduit, was removed. To allow later valve interventions, RVOT was enlarged using a bovine patch. This made the surgical implantation of a larger stented bioprosthesis possible (Fig 1). In cases were operation was performed in beating heart technique (n = 25 (31.6%)), bicaval cannulation was used to achieve a total bypass. Transesophageal echocardiography was used for left heart bubble control during surgery and to ensure, that there was no shunt present.

### Surgical data

Median duration of surgery was 283 (342–221) minutes. In 25 patients (31.6%), beating heart technique was used. Median primary extracorporeal circulation (ECC) time was 164 minutes (203–124). If surgery was performed in cardioplegic cardiac arrest, median primary X-clamp time was 91 (114.5–68) minutes. In 3 patients (3.8%) a second X-clamping was necessary. Mainly blood cardioplegia was used (n = 43, 5.4%) and applied antegrade (n = 36, 45.6%). Surgery was performed in mild hypothermia (33.5 (36–32) °C).

In 74 cases (93.7%), a stented bioprosthesis (PERIMOUNT, Edwards Lifesciences Corporation, Irvine, California, U.S.) was implanted. Large valve diameters (25 mm) were preferred in order to provide a basis for future valve-in-valve interventions (n = 62, 78.5%).

**Fig 2:**
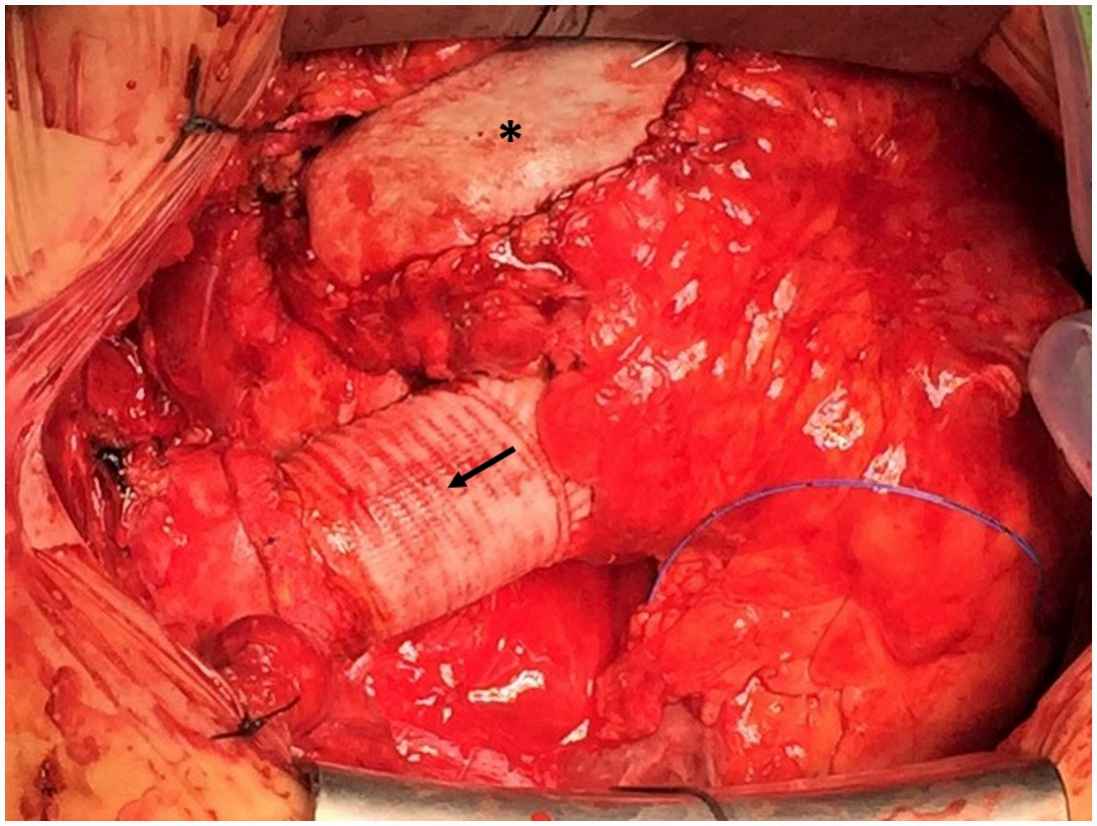
Surgical result in a patient with pulmonary valve replacement (patch-enlarged RVOT marked with asterisk) and concomitant replacement of the ascending aorta (marked with arrow) (surgeons’ view).

In 45 cases (57%) concomitant surgery was performed: tricuspid valve surgery (n = 28, 35.4%), atrial septal defect closure (n = 9, 11.4%), aortic valve replacement (n = 8, 10.1%), coronary artery bypass surgery (n = 6, 7.6%), replacement of the ascending aorta (n = 4, 5.1%), ventricular septal defect closure (n = 4, 5.1%), mitral valve surgery (n = 2, 2.5%), correction of pulmonary vein malformations (n = 2, 2.5%), Glenn operation (n = 1, 1.3%), aortic root reconstruction (n = 1, 1.3%), epicardial ablation (n = 1, 1.3%) and pacemaker probe removal (n = 1, 1.3%). Fig 2 shows the surgical result in a patient with pulmonary valve replacement and concomitant replacement of the ascending aorta. Surgical data is summarized in Table 2.

**Table 2:** Surgical data.

Surgical Data	n (%) or median (IQR3-1)
Duration of surgery [min]	283 (342–221)
Beating heart technique	25 (31.6)
Primary ECC time [min]	164 (203–124)
Primary X-clamp time [min]	91 (114.5–68)
Blood cardioplegia	43 (54.4)
Antegrade cardioplegia application	36 (45.6)
Reperfusion time [min]	32.5 (47–22.3)
Body temperature during surgery [°C]	33.5 (36–32)
Stented bioprosthesis (PERIMOUNT)	74 (93.7)
25 mm prosthesis	62 (78.5)
Conduit	
• Medtronic Freestyle^®^ prosthesis	3 (3.8)
• Homograft	2 (2.5)
Concomitant surgery	45 (57)
• TVS	28 (35.4)
• ASD closure	9 (11.4)
• SAVR	8 (10.1)
• CABG	6 (7.6)
• AAR	4 (5.1)
• VSD closure	4 (5.1)
• MVS	2 (2.5)
• CPVM	2 (2.5)
• Glenn	1 (1.3)
• ARR	1 (1.3)
• EA	1 (1.3)
• PPR	1 (1.3)

TVS = tricuspid valve surgery, ASD = atrial septum defect, SAVR = surgical aortic valve replacement, CABG = coronary artery bypass graft, AAR = replacement of ascending aorta, VSD = ventricular septum defect, MVS = mitral valve surgery, CPVM = correction of pulmonary vein malformations, ARR = aortic root reconstruction, EA = epicardial ablation, PPR = pacemaker probe removal.

### Outcome

**Fig 3:**
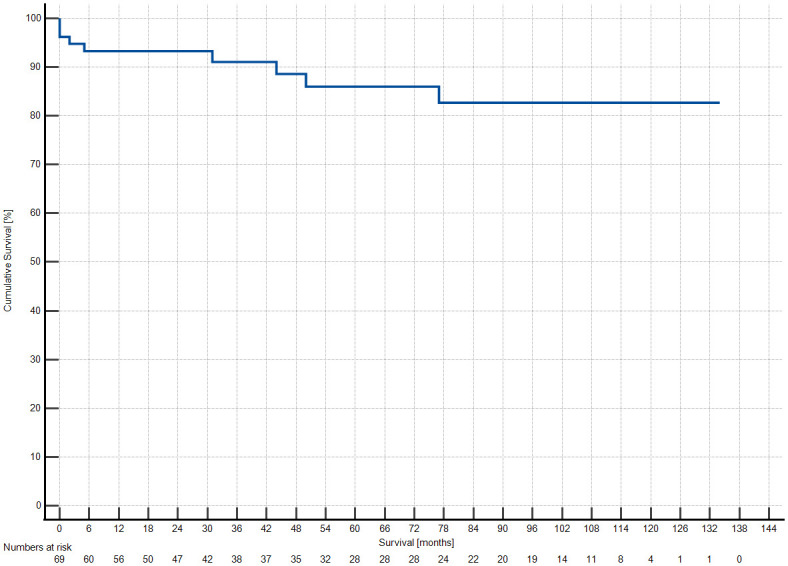
Kaplan-Meier curve showing medium- and long-term follow-up of the patient cohort. Numbers at risk are given below the graph.

In-hospital mortality in our collective was 5.1% (n = 4). 12 patients (15.2%) needed temporary renal replacement therapy after surgery. In 7 cases (8.9%) re-thoracotomy due to bleeding or tamponade was necessary. Deep sternal wound infection (DSWI) incidence was low (n = 1, 1.3%). Low cardiac output occurred in 3 patients (3.8%). 2 patients (2.5%) required an assist device. In 6 (7.6%) patients reintubation was necessary. 10 patients underwent tracheotomy (12.7%). Median ventilation time after operation was 7 hours (15.5–4). A detailed list of post-operative complications is given in Table 3. One year mortality was at 7% (number at risk: 56), five year mortality was 14% (numbers at risk: 28). Kaplan-Meier analysis is depicted in Fig 3.

**Table 3:** Outcome.

Outcome	n (%) or median (IQR3-1)
In-hospital mortality	4 (5.1)
DSWI	1 (1.3)
Bronchopulmonary infection	12 (15.2)
Vein catheter infection	3 (3.8)
Urinary tract infection	5 (6.3)
Bacteremia/sepsis	7 (8.9)
Wound revision	4 (5.1)
Renal replacement therapy	12 (15.2)
Re-thoracotomy due to bleeding/tamponade	7 (8.9)
Low cardiac output	3 (3.8)
Assist device	2 (2.5)
Delirium	6 (7.6)
Ventilation time (h)	7 (15.5–4)
Reintubation	6 (7.6)
Tracheotomy	10 (12.7)

### Post-operative echocardiography upon discharge

Echocardiographic data was available from 89.9% (n = 71) of patients. None of these patients displayed moderate or severe insufficiency upon discharge. Median maximum velocity of the pulmonary valve (V_max_) was 2 m/s (2.3–1.77 m/s).

### Pre- and post-operative MRI data

Both pre- and post-operative MRI data was available from 27.5% (n = 22) of patients. Pre-operative median RV-EDV was 322.5 (370–242) ml compared to 189 (235–172) ml post-operatively (p = 0.0005). Post-operative RV-ESV (113 (126–83) ml) was also significantly lower than pre-operative RV-ESV (179.5 (198–118) ml) (p = 0.0011). These differences remained statistically significant when indexing volume to body surface area (BSA, [m^2^]): Median pre-operative indexed RV-EDV was 183.6 (202.2–131.3) ml/m^2^ compared to median post-operative indexed RV-EDV with 106.9 (122.1–92) ml/m^2^ (p = 0.0006). Median pre-operative indexed RV-ESV was 90.9 (117.3–71.1) compared to median post-operative indexed RV-ESV with 60.4 (64.2–47.6) (p = 0.0017). No significant change was found in RV-EF (p = 0.7859).

## Discussion

Due to major advances in both diagnosis and treatment options of congenital heart defects, which used to have lethal consequences in earlier times, most affected individuals reach adulthood nowadays [6]. Therefore, the cardiac care of ACHD patients has become increasingly important in recent years and specialized care centers have been established.

As therapeutic options for pulmonary valve replacement (PVR) continue to evolve and technologies advance, individual treatment strategies, developed through a collaboration of heart surgeons and (pediatric) cardiologists, become increasingly important. In view of a raising number of ACHD patients, a lifelong strategy to minimize necessary re-do procedures should be the common goal [7].

In sPVR, pericardial bioprostheses have delivered satisfying short- and long-term results [8, 9]. Due to the low pressure physiology of the right heart, correction with mechanical valves (MV) should be avoided. MV carry a high risk of valve thrombosis [10]. Additionally, a bioprosthesis can provide the basis for future valve-in-valve interventions as part of a long-term treatment strategy. From this background, we mainly used stented bovine pericardial bioprostheses (Perimount) with large diameter (25 mm) for pulmonary valve replacement in our cohort of patients.

Our described surgical technique involving a patch enlargement of the RVOT allows a standardized valve implantation comparable to the technique used for surgical aortic valve replacement (sAVR). An advantage of sPVR can also be seen in the performance of concomitant surgery. In our cohort, this was necessary in 57% of cases. This certainly contributed to the relatively long ECC time (164 minutes (203–124)) that can also be explained by peripheral cannulation before sternal re-entry.

Previously conducted studies have shown low mortality rates for sPVR after Fallot correction: An analysis of 21 patients receiving sPVR after Fallot correction showed a 30-day-mortality of 0%. Their cohort was much younger and concomitant surgical procedures were only performed in 28.6% of cases [11]. In another younger collective of 131 patients (14.8 vs. 42 years) who had previously undergone TOF repair, Jang et al. reported no early or late deaths [12]. Lee et al. assessed the outcomes of 61 patients after redo sPVR. They reported a 5-year-survival rate of 94.8% and a 10-year-survival rate of 83.7% [13]. In a meta-analysis of 48 studies, including 3,118 patients pooled 30-day mortality of PVR after operative repair of TOF was 0.87% and pooled 5-year mortality was at 2.2% [3]. We observed mortality rates of 5.1% (in-hospital), 7% (one year follow-up) and 14% (five year follow-up).

In light of different mortality rates, one should take a closer look at risk factors for mortality. For this purpose Jain et al. conducted a multivariate analysis and identified an age greater than 40 years (odds ratio 9.89) and concomitant surgery (odds ratio 6.65) as risk factors for mortality after sPVR in adults with previously corrected ToF [14]. In the wake of 57% (n = 45) concomitant operations and a median patient age of 41.7 (52.2–28.8) years, the in-hospital mortality rate of 5.1% (n = 4) in our cohort can be explained.

Post-operative echocardiographic data showed excellent short-term results with no moderate or severe insufficiency upon discharge. This rate is in line with results reported in literature [15].

Significantly decreased RV-ESV and EDV have similarly been reported in previously conducted MRI studies [16]. No significant RV-EF differences are also in line with literature, as regurgitation and shunting were not taken into account during calculation [16].

Different operative techniques have been proposed for sPVR. The common basis is seen as an implantation of a large diameter bioprosthesis [17]. Our surgical approach underlines this aspect, as we mainly used 25 mm bioprostheses.

## Conclusion

Our study shows that SPVR with our surgical approach, can be performed safely, with an acceptable in-hospital mortality rate and satisfying post-operative results in ACHD patients. It can serve as a solid therapeutic option for patients who may be in need of future valve-in-valve interventions.

## Limitations

This study is a single-center retrospective analysis. Multi-center collaboration can help to enhance the generalizability of the findings in the future. The analysis mainly focusses on short-term post-operative results. Long-term data on valve function, thrombosis, infection rates, and the need for reintervention remains to be assessed in future studies. The MRI study reflects only a small fraction of the study population. This is mainly due to inconsistent indications and collected parameters for MRI. Therefore, generalizations should be drawn with caution. To increase the MRI follow-up rate in future studies, standardized protocols for ACHD patients undergoing sPVR are desirable.

## Supporting information

S1 File Supplementary data. (XLSX)
